# Engineering the mechanical characteristics of regenerated silk fibroin materials: the impact of chemical and physical modification strategies

**DOI:** 10.3389/fchem.2025.1606995

**Published:** 2025-06-10

**Authors:** Haitao Guan, Fang Ding, Ye Xue, Jinli Zhao

**Affiliations:** ^1^ Clinical Innovation Research Center of Nantong University-Nantong Hospital of Traditional Chinese Medicine, Nantong University, Nantong, Jiangsu, China; ^2^ Department of Pain and Ultrasonography, Nantong Hospital Affiliated to Nanjing University of Chinese Medicine, Nantong, Jiangsu, China; ^3^ Department of Imaging, Nantong Hospital Affiliated to Nanjing University of Chinese Medicine, Nantong, Jiangsu, China; ^4^ School of Life Sciences, Nantong University, Nantong, Jiangsu, China; ^5^ Department of Imaging, Affiliated Hospital of Nantong University, Nantong, Jiangsu, China

**Keywords:** regenerated silk fibroin, mechanical property, plasticizer, chemical modification, natural polymer

## Abstract

In this study, an in-depth exploration was conducted on the mechanical properties of regenerated silk fibroin (RSF) materials through diverse processing modalities, encompassing wet pressing, humidity treatments at varying levels, and the incorporation of plasticizers. Notably, these approaches induced substantial modifications in the mechanical properties of RSF materials. The elastic strength of RSF exhibited a wide range, from 12.6 to 1,644.8 MPa; the ultimate strength spanned from 0.12 to 42.63 MPa, and the maximum elongation at break fluctuated between 0.67% and 614.38%. Additionally, the mechanisms underlying the effects of these distinct treatment methods were meticulously investigated. This fundamental research not only provides crucial insights into the modulation of silk fibroin’s mechanical properties but also holds significant promise for broadening its applications in the biomedical engineering domain, particularly in the pivotal fields of bone tissue and tendon regeneration.

## 1 Introduction

Silk is a natural material famous for its good biocompatibility, and it is a potential substitute for petroleum-based plastics (Altman et al., 2003; Wang et al., 2006; Zheng et al., 2022). Silk materials have been manufactured into various biomedical devices, such as bone screws, artificial blood vessels, and drug delivery carriers. Physical properties are an important factor that influences the applications of silk materials. Common physical properties include strength, elasticity, toughness, topography, and conductivity. Based on the specific applications, physical and chemical properties of silk have been well studied. For instance, secondary structures of silk fibroin (SF) have been characterized and modified to control the drug release rate and degradation rate (Choi et al., 2018; Long et al., 2022; Pritchard and Kaplan, 2011). However, regenerated silk fibroin (RSF) materials still face the challenge of poor mechanical properties. The RSF made by the formic acid method is rigid and stiff in the dry state, while the RSF made by the water-based method is fragile. This limits its applications in many fields.

The silk fiber is a natural fiber material with excellent mechanical properties. Silk fibers perform well in tensile strength (1–4 GPa), toughness, elasticity [elastic modulus (stiffness) is approximately 10–15 GPa], and elongation at break (up to 20%–30%), all of which can be comparable to those of steel with the same quality (Babu, 2017; Qiu et al., 2019; Su and Buehler, 2016; Wu et al., 2019). Therefore, silk fibers have been used as textile materials to produce high-strength fabrics. From a microscopic perspective, a piece of silk is mainly composed of sericin and silk fibroin. Sericin is wrapped in the outermost layer of silk fibroin, which plays a role in adhering to and protecting silk fibroin fibers (Ma et al., 2024; Ma et al., 2024). The main component of sericin contains a large amount of serine and other amino acids, which contribute to its good water solubility and viscosity (Cao and Zhang, 2016; Shen et al., 2022). Silk fibroin accounts for the vast majority of a piece of silk. Silk fibroin is mainly composed of serine (Serine), glycine (Glycine), and proline (Prolin), accounting for more than 70% (Koh et al., 2015; Wahab et al., 2019; Warwicker, 1954). Other amino acids such as valine (Leucine), glutamic acid (Glutamic acid), and alanine (Alanine) also exist in silk fibroin, but the proportions are low (Chen et al., 2015; Nadiger et al., 1985). Silk usually needs to be degummed first to remove the sericin to obtain high-purity silk fibroin for use as biomedical materials (Xue et al., 2019). There are several reasons for this practice. First, sericin serves as a protective layer for silk fibers. There are many impurities, and the purity is not high. Second, sericin has poorer biocompatibility than silk fibroin and may cause immune rejection. Third, sericin has lower plasticity than silk fibroin, and its mechanical properties are also weaker (Wang et al., 2023). Therefore, silk fibroin has attracted widespread attention as a good biomaterial. Its physicochemical structure has been well studied. Silk fibroin has a highly ordered secondary structure, including an α-helix, a β-sheet, and a β-turn (Hu et al., 2006). The secondary structures that have received the most attention are β-sheets and α-helices, and the performance of regenerated silk fibroin materials largely depends on their quantity and distribution (Drummy et al., 2005; Zhang et al., 2020). From the above analysis, the high strength and high toughness of silk fiber are due to the unique core-shell structure of the silk fiber, where the silk fibroin and sericin function as core and shell, respectively. The secondary structure of silk fibroin fibers, especially the tight β-sheet structure, provides sufficient strength to silk fibers. The presence of sericin enables the silk fiber to stretch and absorb energy when subjected to external force, making it less prone to breaking.

However, regenerated silk materials (RSMs) almost totally consist of silk fibroin, which can easily experience phase transition from α-helix to β-sheets when exposed to water or other organic solvents (Hu et al., 2008; Jain et al., 2023; Nandakumar et al., 2020). This enables the properties of the regenerated silk materials to be arbitrarily controlled, but another problem arises as the regenerated silk materials tend to be brittle or rigid. After regeneration, the RSM loses the core-shell structure and the unique combination of crystalline and amorphous regions of silk fiber. This can be one reason why RSM is rigid and hard. In silk fibers, the outer layer of sericin can not only absorb the tensile stress but also preserve some water molecules, which function as plasticizers. Another reason is the relatively high crystallinity (up to 45%) of RSM, which restricts the mobility of the protein chains and limits the material’s flexibility (Chelazzi et al., 2020; Lu et al., 2015). The native tissues of the human body usually have a very low elastic modulus; for example, sciatic nerve tissue is 0.6 MPa, and muscle tissue is 0.02 MPa (Hoang et al., 2020; Liu et al., 2023). Therefore, tissue engineering materials also need to be very soft to avoid damaging soft tissues during repeated deformation and displacement. It is necessary to systematically explore a method to modify the mechanical properties of RSM.

Blending materials is a common and useful method to modify the properties of materials. By blending several different components, the drawbacks of the materials can be fixed while the desirable properties can be achieved. Silk fibroin mainly consists of glycine, alanine, and serine in different percentages, and it is rich in polar groups such as carboxyl and hydroxyl groups. These polar groups can be functionalized with their counterparts from additives. In addition, the intermolecular interactions formed between the matrix material and the additives, such as hydrogen bonds, van der Waals forces, entanglements between different components, and the recombination between the crystalline and amorphous phases caused by the additives, can change the mechanical properties of the composite materials. In this study, we proposed applying several types of small-molecule chemicals into the silk fibroin matrix and systematically studying the impact of the intermolecular interactions between these chemicals and the silk fibroin matrix on the mechanical properties of silk fibroin composites.

## 2 Materials and methods

### 2.1 Materials and synthesis

**FIGURE 1:**
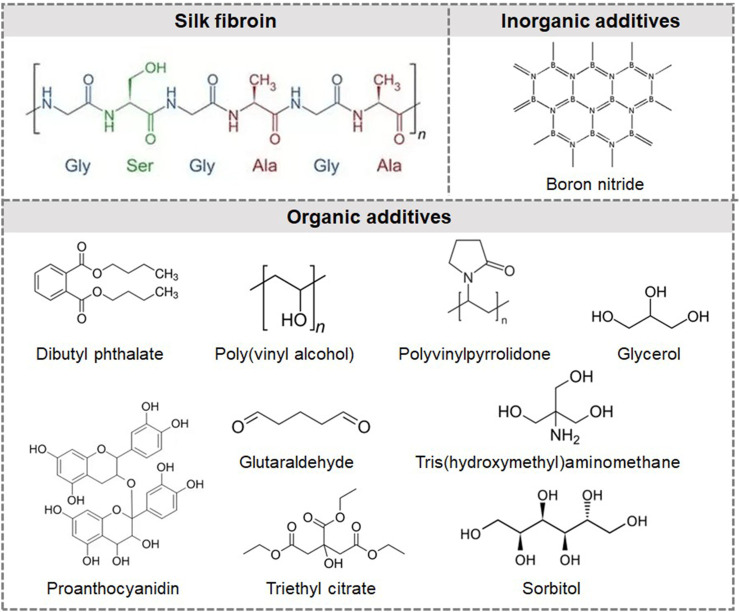
Molecular structures of silk fibroin, BN, proanthocyanidin, polyvinylpyrrolidone, glycerol, dibutyl phthalate, poly(vinyl alcohol), sorbitol, triethyl citrate, and tris (hydroxymethyl) aminomethane.

*Bombyx mori* mulberry (Mori) cocoons were purchased from Xinyuan Sericulture Company (Nantong, Jiangsu, China). Cocoons were degummed first. Silkworms were removed from the cocoons, and the cocoons were cut into small pieces and then boiled in a 0.02 M Na_2_CO_3_ solution for 30 min. The cocoon pieces were totally submerged in the solution during the whole process. At the end of this process, the cocoons were dissociated into SF fibers. Thereafter, the SF fibers were thoroughly washed with DI water thrice to completely remove the sericin coating. SF fibers were dried in a vacuum oven at room temperature for 48 h to remove the remaining moisture. To make regenerated SF composites, the SF fibers were dissolved in a solution of formic acid containing 4 wt% CaCl_2_ at ambient temperature. A certain amount of silk fibroin solution was poured onto the self-designed PDMS mat and then allowed to naturally dry in a fume hood for 48 h to obtain a pure silk fibroin membrane (Mori film). The reproduced Mori film is prepared by dissolving the regular Mori film into the formic acid solution again to evaluate the influence of formic acid on the mechanical properties of the Mori film (reproduced Mori film). The water-soaked Mori film was prepared by soaking a piece of Mori film (circle shape with a diameter of 5 cm and a thickness of approximately 0.5 cm) in distilled water for 30 min, then allowing it to dry completely to evaluate the influence of water on the mechanical flexibility of the Mori film (water-soaked Mori film). The methanol-soaked Mori film was prepared by soaking a piece of regular Mori film in a methanol solution for 30 min and then drying the film in a fume hood to evaluate the influence of methanol on the mechanical flexibility of the Mori film (methanol-soaked Mori film). To compare the difference between composite films with organic and inorganic additives, boron nitride (BN), dibutyl phthalate (DBP), glutaraldehyde, glycerol, polyvinylpyrrolidone (PVP), proanthocyanidin, polyvinyl alcohol (PVA), sorbitol, triethyl citrate, and tris(hydroxymethyl)aminomethane were selected and mixed with Mori silk solution to obtain composite films. All these chemicals were used as received. The weight ratios of BN are 1%, 5%, 10%, and 25%. All organic plasticizers were used at a weight ratio of 20%, except for PVA, which was used at both 20% and 30%. The molecular structures of the silk fibroin, inorganic additives, and organic additives are shown in Figure 1.

To study the influence of moisture on the mechanical properties of silk films, three groups of Mori films (nine pieces in total) with similar thickness (approximately 0.6 mm) were first soaked in DI water for 24 h, ensuring that all CaCl_2_ could completely be removed. Then, water on the surface of the soaked Mori films was wiped out, and all the samples were kept in a vacuum chamber under a 30 in Hg vacuum for 72 h. First, three pieces of samples were characterized using the SHIMADZU Universal/Tensile Testing machine. Then, three other pieces of the samples were kept in a humidity chamber with humidity controlled at 50%, and the remaining three pieces were kept in a humidity chamber with humidity controlled at 70% for a long enough time. After reaching the equilibrium, all six samples were also characterized using the SHIMADZU Universal/Tensile Testing machine.

To prepare the fixed, hot-pressed, rolled, and uncompressed films, formic silk solutions were first prepared. The silk fibroin films are dissolved in a CaCl_2_ solution, yielding a 15% (w/v) solution, and then cast onto 3.2″ × 4.9″ PE rectangular molds and dried. The films were then soaked in water for 24 h to remove CaCl_2_ and pat-dried with a towel to remove excess water.

For the film fixing method (FFM) processing, the films were cut into appropriately sized rectangular and dog-bone specimens with a width of 12.5 mm and a gauge length of 40 mm before fixing the specimens in the x- or y-direction. The film-fixing apparatus was made previously for post-stretched SF films in both directions. The apparatus consisted of die-cut aluminum plates that were constructed into a spring-loaded clamp device to allow flat mirror facets to grip and fix films on all sides (biaxial planar directions) or along one axis (uniaxial). The film is pre-stretched on a square frame, providing tension to all sides, resisting shrinkage when dried. In this study, the film-fixing apparatus was used to direct and fix a soaked and crystalizing SF film in a single direction (x- or y-direction). For single-directional planar stretching, at least one aluminum clamp is attached to one side of a rectangular SF polymer film, and a second clamp is attached to another end parallel to the single planar stretch direction. The spring-loaded clamps are then fixed on adjustable hooks in a pre-stretched state, which allows the pre-stretched SF film to expand and remain taut and flat while suspended during drying. A pair of mechanical clamps, coupled to each end of a square frame with loaded springs, allows SF films to be pre-stretched, remaining flat initially, and post-stretched as the film dries, resulting in a unidirectionally tensioned film. The fixed-stretched polymer films have thicknesses up to 6 mm. Mechanical tests were performed to monitor the strain feedback for the one single-direction stretching as the fixed pre-stretched polymer film is contracting. In addition, mechanical tests were also performed in the opposite direction of the non-stretched side on the biaxial planar direction to observe changes in the crystallization alignment.

### 2.2 Characterization

Silk fibroin composites were cut into tensile specimens of dog-bone shape according to ASTM D-3039. Uniaxial tension tests were performed using a computer-controlled AGS-10KN Universal test machine (Shimadzu Corporation, Japan) under ambient conditions. A constant crosshead speed of 2 mm/min was specified with different load cells. An extensometer was used to monitor the specimen’s longitudinal strain. Instantaneous load and displacements were simultaneously recorded. From the force–displacement curves of five specimens with the same fiber content, the mean tensile modulus E, tensile strength σu, and elongation ɛu were calculated.

The surface morphologies of all SF films blended with plasticizers were characterized by LEO 1530 VP SEM at Nantong University. Each sample was cut into rectangular pieces with a length between 5 and 10 mm and was coated with a thin layer of Au film. The thickness of the Au film is approximately 200 Å. Once coated, the samples were ready for SEM imaging. The magnification of the samples is 1000×.

FT-IR analysis was conducted using a Bruker FT-IR Spectrometer Tensor 27 (Bruker Co. Ltd., Billerica, MA, United States), equipped with a deuterated triglycine sulfate detector and a multiple-reflection, horizontal MIRacle attenuated total reflection (ATR) attachment (using a Ge crystal). The analysis was set up with 128 scans at a resolution of 4 cm^−1^. The wave number of each sample ranges from 400 to 4,000 cm^−1^.

The thermal property of each sample was evaluated using a TA Instrument Q100 DSC (New Castle, DE, United States). The sample was encapsulated in an aluminum pan with a weight ranging from 5 mg to 10 mg. The sample was heated along with a purged dry nitrogen gas flow of 50 mL/min and equipped with a refrigerated cooling system. All the measurements were performed at a heating rate of 2°C/min.

## 3 Results and discussion

### 3.1 Properties of regular and reproduced mori films

**FIGURE 2:**
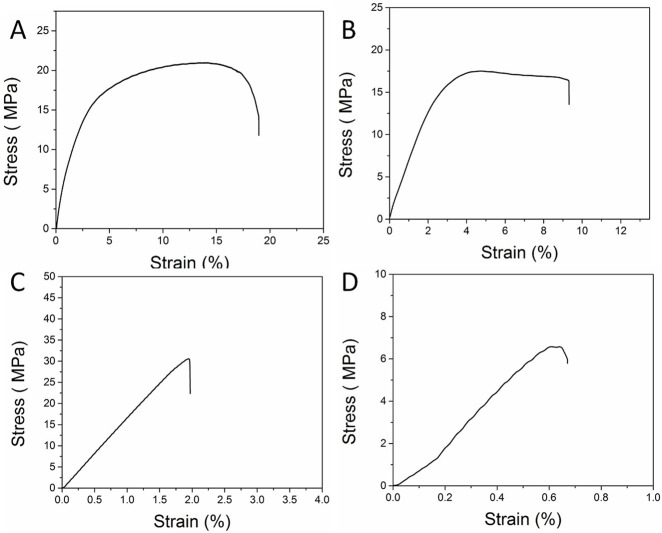
Elastic modulus, strength of extension, and elongation ratio of the **(A)** Mori film, **(B)** reproduced Mori film, **(C)** water-soaked Mori film, and **(D)** methanol-soaked Mori film.

As shown in Figure 2A and Table 1, the maximum elongation at break of regular Mori films is 19.0%, and its elastic modulus is 787.9 MPa. As shown in Figure 2B, the maximum elongation at break of reproduced Mori films decreased to 9.3%, and their elastic modulus was 667.5 MPa. Compared with those of the original Mori film, both the elongation and elastic modulus of reproduced Mori films decreased, which can be attributed to the lower molecular weight of silk protein fibers caused by the influence of formic acid. Therefore, the reproduced Mori film is softer and more flexible. The stiffness of Mori films is increased after being soaked in either water or methanol solution. This increase could be attributed to the high crystallinity obtained after being processed in water or methanol solution. As shown in Figure 2C, the maximum elongation at break of water-soaked Mori films is 1.97%, and its elastic modulus is 1.645 GPa, which is much higher than that of the original Mori film. It can be concluded from Figure 2D that the methanol-soaked Mori film has a very high elastic modulus, 1.093 GPa, but its maximum elongation at break decreases to 0.67%. Water and methanol soaking induce distinct structural reorganization mechanisms in RSF films. Water acts as a plasticizer by disrupting hydrogen bonds between silk fibroin chains, increasing molecular mobility. During drying, the evaporation of water triggers the β-sheet formation through hydrophobic interactions and hydrogen bond reconfiguration. This results in increased crystallinity and a higher elastic modulus (Hu et al., 2011; Lu et al., 2009). The β-sheet-dominated structure enhances stiffness but reduces ductility.

**TABLE 1:** Mechanical properties of various Mori film samples.

Samples	Maximum elongation at break (%)	Yield strength (MPa)	Ultimate strength (MPa)	Elastic modulus (MPa)
Mori film	19.0 ± 0.6	6.55 ± 0.3	21.02 ± 1.1	787.9 ± 12.4
Reproduced Mori film	9.3 ± 0.2	11.08 ± 1.1	17.54 ± 0.9	667.5 ± 11.2
Water-soaked Mori film	1.97 ± 0.1	27.55 ± 1.6	30.69 ± 2.3	1644.8 ± 14.5
Methanol-soaked Mori film	0.67 ± 0.03	6.56 ± 0.5	6.67 ± 0.4	1093.4 ± 12,4

### 3.2 Effects of hydroxyl plasticizers

**FIGURE 3:**
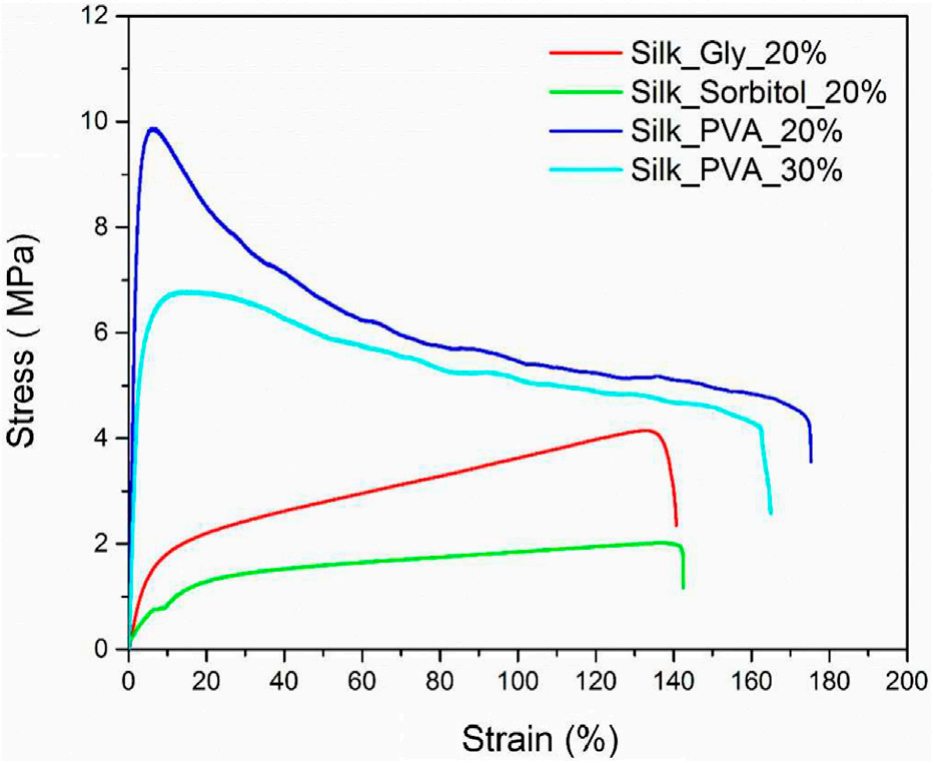
Stress–strain curves of Silk_Gly_20%, Silk_Sorbitol_20%, Silk_PVA_20%, and Silk_PVA_30%. All these percentages are based on the weight percent between the plasticizer and silk.

Hydroxyl-rich plasticizers modulate RSF mechanics via hydrogen bond competition. Hydroxyl groups (−OH) form hydrogen bonds with silk fibroin’s carboxyl (−COOH) and amide (−CONH−) groups, weakening inter-chain interactions. This reduces crystallinity and enhances chain mobility. As shown in Figure 3 and Table 2, the maximum elongation at break of the Silk_Gly_20% film is approximately 139.9%, and its elastic modulus is approximately 34.5 MPa. The maximum elongation at break of the Silk_Sorbitol_20% film increased to 142.4%, and its elastic modulus was 12.6 MPa. The maximum elongation at break of the Silk_PVA_20% film increased to 175.2%, and its elastic modulus was 406.3 MPa. Compared with those of the original Mori film, both the elongation and elastic modulus of reproduced Mori films decreased, which could be attributed to plasticizers. All three plasticizers are present at 20 wt% relative to the silk in the film. Among these three films, Silk_Sorbitol_20% is the most flexible sample, which is 12.6 MPa. However, the ultimate strength of the Silk_Sorbitol_20% samples is the lowest, i.e., 2.05 MPa. All three types of plasticizers have a high percentage of hydroxyl groups, contributing to their hygroscopic properties. However, compared with glycerol and sorbitol, which are all small molecules, PVA is a synthetic polymer with a much higher molecular weight. PVA is an atactic polymer that exhibits crystallinity, and it has excellent forming and adhesive properties. PVA itself has high tensile strength and flexibility. The silk and PVA fibers are well blended and entangled together during the fabrication. All of these factors may account for the much higher elongation and elastic modulus of Silk_PVA_20% than those of Silk_Glycerol_20% and Silk_Sorbito_20%. Moreover, the Silk_PVA_30% sample has the highest ultimate strength at 9.88 MPa compared to the other three samples. For the Silk_PVA_30% sample, the elastic modulus decreases to 233.5 MPa, which is much lower than that of the Silk_PVA_20% sample. As PVA contains many hydroxyl groups, which confer polarity and enable hydrogen bonding with water, it exhibits hygroscopic properties. The mechanical performance of silk/PVA composites is governed by the synergistic characteristics of both components. Upon increasing the PVA content, the moisture absorption capacity of the composite membranes significantly intensifies, equivalent to the introduction of water as a plasticizing agent. Notably, as previously noted, elevated water content markedly enhances the flexibility of the silk component. Therefore, it is reasonable to expect that with more PVA added, the composite film will become more flexible. Moreover, small molecules diffuse easily into the fibroin matrix, disrupting crystallinity and enhancing ductility. However, excess low-MW plasticizers may leach out, reducing long-term stability. For high-MW additives, polymers entangle with fibroin chains, forming a semi-interpenetrating network. This increases entanglement density, improving ultimate strength. However, excessive MW restricts chain mobility, reducing the elongation at break.

**TABLE 2:** Mechanical properties of Silk_Gly_20%, Silk_Sorbitol_20%, Silk_PVA_20%, and Silk_PVA_30%.

Samples	Maximum elongation at break (%)	Ultimate strength (MPa)	Elastic modulus (MPa)
Silk_Glycerol_20%	139.9 ± 6.3	4.15 ± 0.2	34.5 ± 2.4
Silk_Sorbitol_20%	142.4 ± 8.2	2.05 ± 0.1	12.6 ± 1.1
Silk_PVA_20%	175.2 ± 9,2	9.88 ± 0.4	406.3 ± 16.7
Silk_PVA_30%%	164.1 ± 8.5	6.78 ± 0.4	233.5 ± 18.3

### 3.3 Effects of inorganic plasticizers (BN)

**FIGURE 4:**
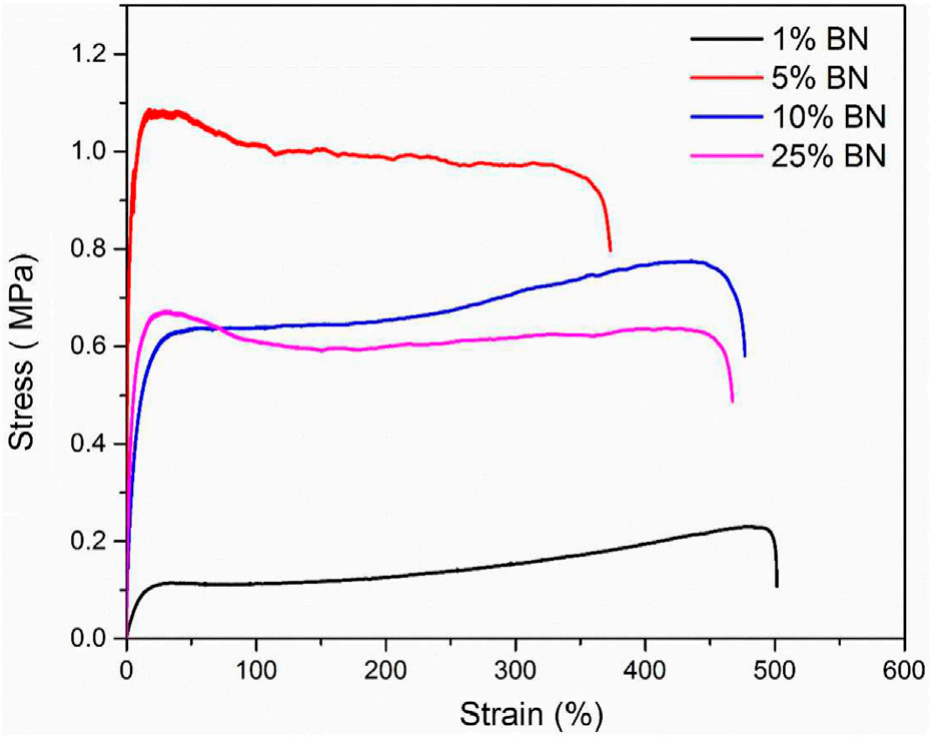
Stress–strain curves of Silk_BN_1%, Silk_BN_5%, Silk_BN_10%, and Silk_BN_25%.

As shown in Figure 4 and Table 3, the maximum elongation at break of the Silk_BN_1% film is approximately 501.5%, and its elastic modulus is approximately 1.02 MPa. The maximum elongation at break of the Silk_BN_5% film decreased to 373%, and its elastic modulus was 102.26 MPa. The maximum elongation at break of the Silk_BN_10% film increased to 476.9%, and its elastic modulus was 19.45 MPa. Compared with that of the original Mori film, the elastic modulus of these four samples decreased. It is worth noting that the silk_BN_5% sample has the highest elastic modulus at 102.26 MPa, which is much lower than that of the other three samples. It is believed that silk-BN composite films with 5 wt% may be well-blended and the inorganic BN additives may distribute homogeneously among the silk fiber networks.

**TABLE 3:** Mechanical properties of Silk_BN_1%, Silk_BN_5%, Silk_BN_10%, and Silk_BN_25%.

Samples	Maximum elongation at break (%)	Ultimate strength (MPa)	Elastic modulus (MPa)
Pure Mori	548.66 ± 21.2	0.12 ± 0.01	0.95 ± 0.05
Silk_BN_1%	501.5 ± 23.5	0.23 ± 0.0.01	1.02 ± 0.08
Silk_BN_5%	373.1 ± 18.5	1.08 ± 0.13	102.26 ± 21.3
Silk_BN_10%	476.9 ± 23.7	0.77 ± 0.04	19.45 ± 1.4
Silk_BN_25%	467.2 ± 26.4	0.67 ± 0.03	50.99 ± 3.8

### 3.4 Effects of different organic plasticizers

Cast and soaked silk films are brittle at room temperature, so plasticizers are added to make them more flexible. The tensile properties of these films are known to be affected by the relative humidity of the ambient air since water acts as a form of plasticizer. However, little is known about how the plasticizers are affected by relative humidity. When SF films are exposed to high humidity, the absorbed water further plasticizes the silk. The type of plasticizers used can determine the extent of the changes observed in the tensile properties of films stored at different humidity levels, which is why we selected a range of hydrophobic and hydrophilic plasticizers. Depending on the plasticizers used in the film, there may be differences in the amount of water absorbed. Since there is a vast and diverse range of plasticizers to choose from, FT-IR analysis was used to scan and test the samples and observe their chemical properties, and a simple physical bend test was performed by hand to prescreen viable plasticizers before performing full tensile, ductility, and soundness analyses, as shown in Supplementary Figure S1 and Supplementary Table S1.

Twelve types of selected plasticizers, namely, Tris (tris(hydroxymethyl)aminomethane), triethyl citrate, triethylene glycol (TEG), PVP, dioctyl phthalate (DOP), oleic acid, glycerol, sorbitol, PVA, proanthocyanidin, dibutyl phthalate (butyl), and glutaraldehyde, were mixed with the Mori silk solution to obtain flexible composite films, all at a weight ratio of 20%. Tris is an organic compound that has been extensively used in biochemistry. Tris can stabilize the pH of a system and inhibit a number of enzymes. It is also used to increase the permeability of cell membranes. However, as shown in Supplementary Figure S2A, Tris and silk proteins are phase-separated on the micrometer scale. The Tris domains are layers of aggregates, and the silk molecules show a uniform surface feature. Triethyl citrate is a colorless liquid used as a surface-active agent. It can be found in Supplementary Figure S2B that triethyl citrate formed aggregates and distributed evenly in the silk protein matrix. TEG is a colorless and odorless viscous liquid and is widely used as a plasticizer for vinyl and a desiccant for gas. As shown in Supplementary Figure S2C, TEG and silk protein are well mixed, and there is no phase separation found. In addition, many voids were observed, indicating that TEG molecules and silk proteins have formed into small particles with a size of up to 4 μm. Those particles are distributed homogeneously in the film. PVP is a water-soluble polymer and is used as a binder in many materials. In Supplementary Figure S2D, it was found that PVP and silk protein are well mixed without phase separation. However, the film exhibited a rough surface compared to the pure silk film. DOP is an organic compound and has been widely used as a plasticizer in PVC products due to its suitable properties and low cost. However, silk protein and DOP cannot be well mixed on the micrometer scale. As shown in Supplementary Figure S2E, the silk–DOP composite exhibits obvious phase separation, and both phases had a smooth surface. Oleic acid is a fatty acid, and its principal use is as a component in many foods. Its sodium salt derivative is also the main component of soap. As shown in Supplementary Figure S2F, silk protein and oleic acid can be well mixed without phase separation. In addition, it exhibits a wrinkled surface compared to the pure silk surface. Moreover, glycerol is a colorless, odorless, and non-toxic liquid. It is mainly used to improve the smoothness of materials during medical and pharmaceutical procedures. Sorbitol is a sugar alcohol that is commonly used as a thickener and moisturizer in beauty products. Poly(vinyl alcohol) is a water-soluble synthetic polymer and has been widely used as a paper additive and textile sizing agent. Proanthocyanidins are a class of polyphenols that are abundant in a variety of plants. DBP is an organic compound that is soluble in various organic solvents. It is also widely used as a plasticizer and an additive. All these five plasticizers can be mixed well with silk proteins, as shown in Supplementary Figure S3A–E. All five composite films exhibit a uniform, smooth surface without visible phase separation. This may account for the good mechanical properties of these films (for example, in our April month report, for silk_PVA_20%, the maximum elongation at break is 175%, the ultimate strength is 9.88 MPa, and the elastic modulus is 406.3 MPa). Finally, glutaraldehyde is used as a disinfectant in the healthcare industry or as a hardener in x-ray film processing. In Supplementary Figure S3F, the silk–glutaraldehyde composite film shows a uniform surface with some cracks, indicating that the composite film is not as mechanically stable as the pure silk film.

**FIGURE 5:**
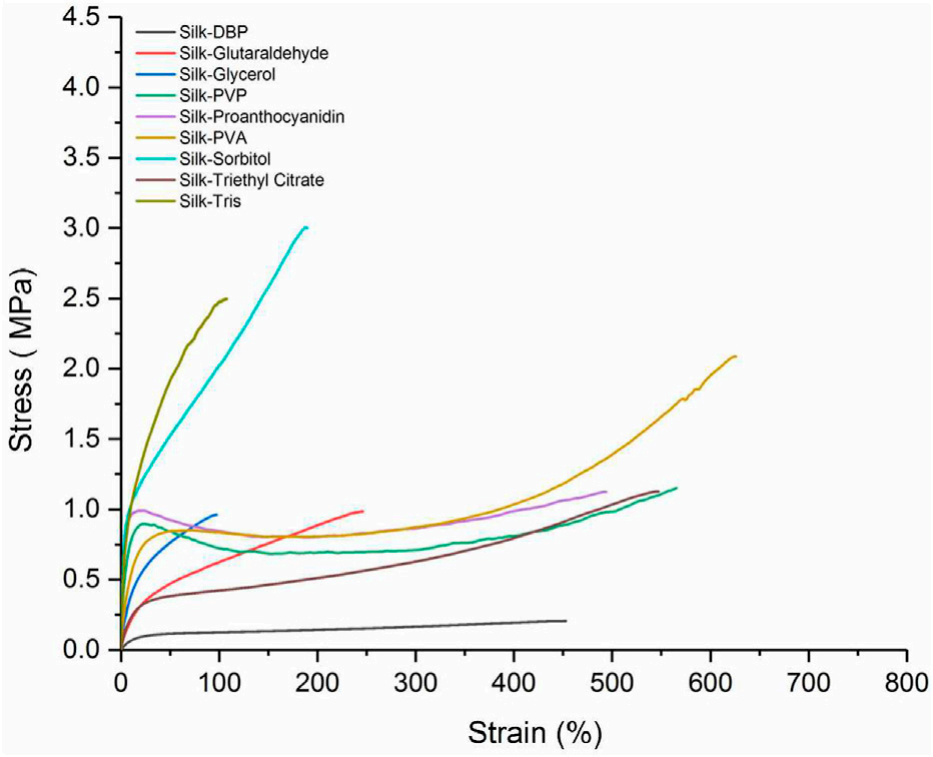
Stress–strain curves of silk–dibutyl phthalate (DBP), silk–glutaraldehyde, silk–glycerol, silk–polyvinylpyrrolidone (PVP), silk–proanthocyanidin, silk–poly(vinyl alcohol) (PVA), silk–sorbitol, silk–triethyl citrate, and silk–tris(hydroxymethyl)aminomethane.

As shown in Figure 5 and Table 4, the maximum elongations at break of the composite films are in the following order: silk–proanthocyanidin (614.38%), silk–PVA (603.13%), silk–PVP (594.18%), silk–glutaraldehyde (253.43%), silk–DBP(421.64%), silk–triethyl citrate (547.01%), silk–sorbitol (141.48%), silk–Tris (124.15%), and silk–glycerol (97.58%). The ultimate strengths of these composite films are in the following order: silk–sorbitol (2.61 MPa), silk–Tris (2.53 MPa), silk–PVA (2.07 MPa), silk–DBP (1.75 MPa), silk–PVP(1.21 MPa), silk–triethyl citrate (1.13 MPa), silk–glutaraldehyde (1.11 MPa), silk–proanthocyanidin (0.99 MPa), and silk–glycerol (0.96 MPa). The elastic moduli of these composite films are in the following order: silk–sorbitol (27.99 MPa), silk–Tris (20.57 MPa), silk–proanthocyanidin (16.85 MPa), silk–PVP (11.8 MPa), silk–glycerol (6.98 MPa), silk–PVA (7.22 MPa), silk–glutaraldehyde (3.1 MPa), silk–triethyl citrate (2.31 MPa), and silk–DBP (1.18 MPa).

**TABLE 4:** Mechanical properties of silk–dibutyl phthalate (DBP), silk–glutaraldehyde, silk–glycerol, silk–polyvinylpyrrolidone (PVP), silk–proanthocyanidin, silk–poly(vinyl alcohol) (PVA), silk–sorbitol, silk–triethyl citrate, and silk–tris(hydroxymethyl)aminomethane composite films, including elastic modulus, ultimate strength, and maximum elongation at break.

Samples	Maximum elongation at break (%)	Ultimate strength (MPa)	Elastic modulus (MPa)
Silk–DBP	421.64 ± 43.1	0.175 ± 0.04	1.18 ± 0.12
Silk–glutaraldehyde	253.43 ± 36.7	1.11 ± 0.25	3.1 ± 0.41
Silk–glycerol	97.58 ± 11.3	0.96 ± 0.1	6.98 ± 0.8
Silk–PVP	594.18 ± 51.5	1.21 ± 0.24	11.8 ± 3.88
Silk–proanthocyanidin	614.38 ± 262.02	0.99 ± 0.30	16.85 ± 6.79
Silk–PVA	603.13 ± 24.29	2.07 ± 0.13	7.22 ± 1.31
Silk–sorbitol	141.48 ± 41.89	2.61 ± 0.36	27.99 ± 4.93
Silk–triethyl citrate	547.01 ± 45.3	1.13 ± 0.1	2.31 ± 0.14
Silk–Tris	124.15 ± 19.39	2.53 ± 0.06	20.57 ± 0.79

It is also noticeable that the ultimate strength of silk composite films with inorganic additives is extremely low and much less than that of silk composite films with organic plasticizers. However, it is very interesting that the elongation of the silk composite with inorganic additives is generally much higher than that of several organic plasticizers. The effects of different types of organic additives on the mechanical properties of silk fibroin composite membranes need to be further analyzed together with their molecular structural formulas.

### 3.5 Effects of environmental humidity

**FIGURE 6:**
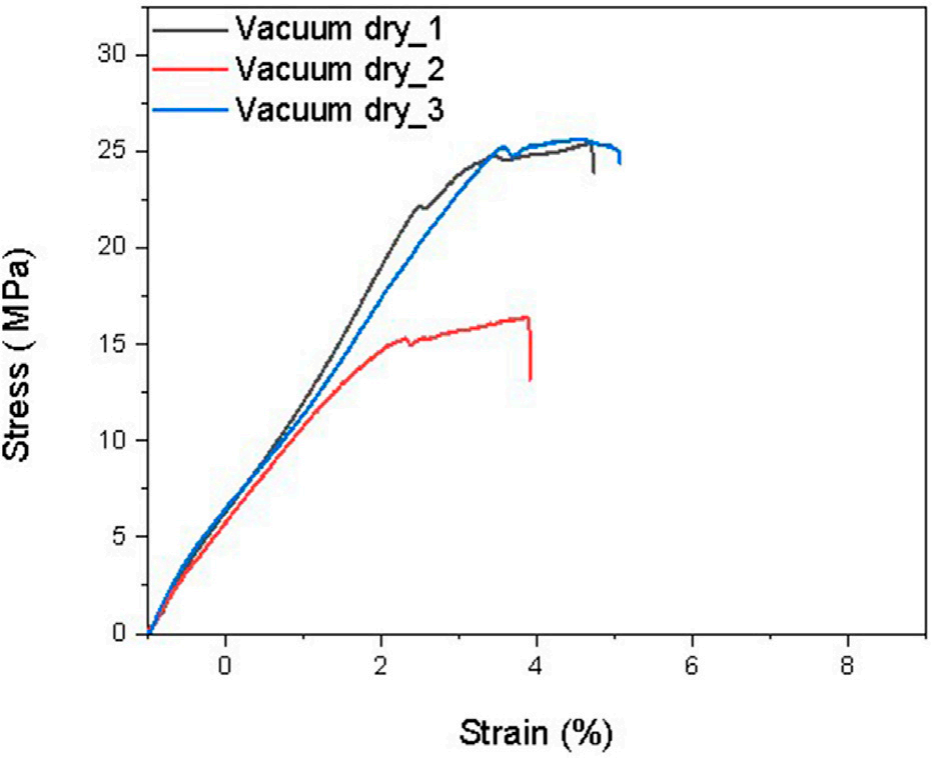
Stress–strain curves of three Mori films after reaching the equilibrium in the vacuum chamber with 30 in Hg vacuum.

**FIGURE 7:**
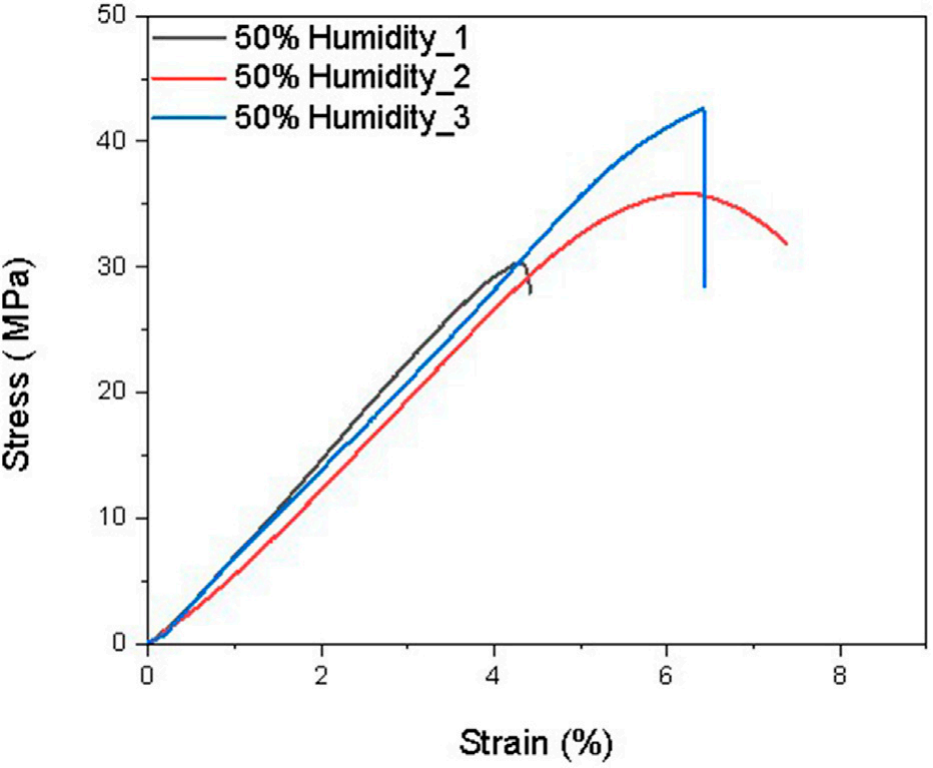
Stress–strain curves of three Mori films after reaching the equilibrium in the humidity chamber with 50% humidity.

**FIGURE 8:**
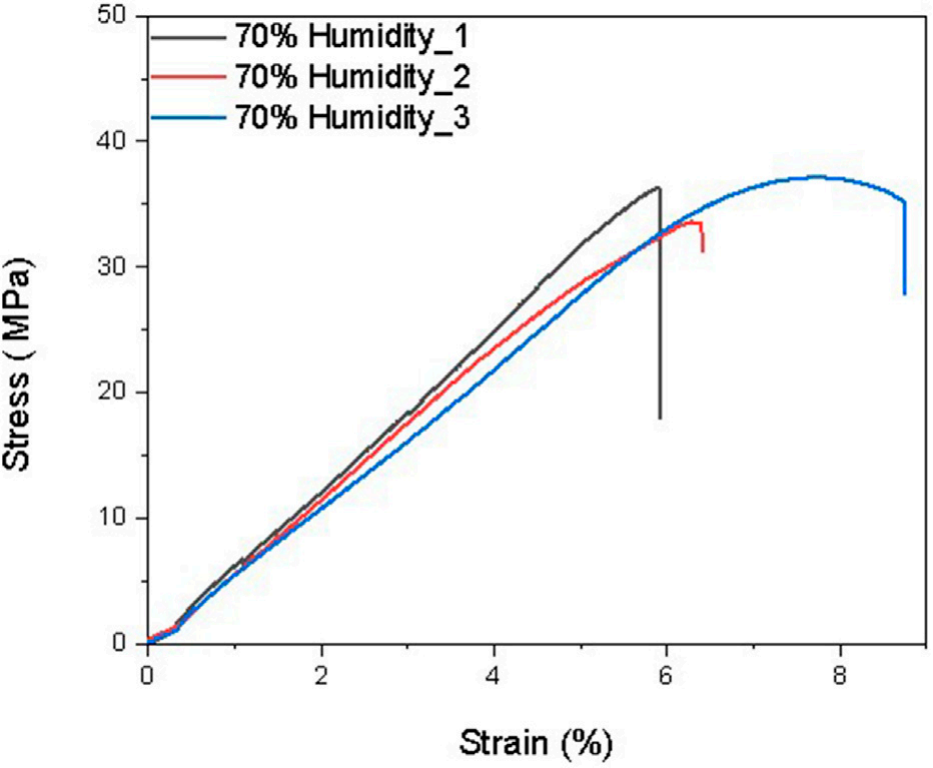
Stress–strain curves of three Mori films after reaching the equilibrium in the humidity chamber with 70% humidity.

**FIGURE 9:**
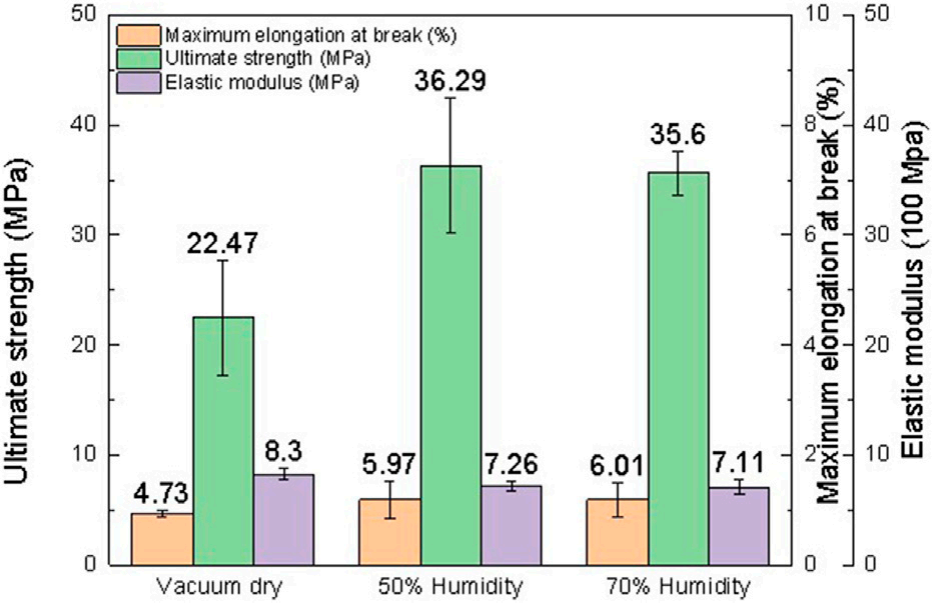
Mechanical properties of Mori films after reaching the equilibrium in the vacuum chamber with 30 in Hg vacuum and the humidity chamber with 50% and 70% humidity, respectively, including elastic modulus, ultimate strength, and maximum elongation at break.

The elastic modulus is a measure of a film’s resistance to elastic deformation when stress is applied. The elastic modulus value is defined as the slope of its stress–strain curve in the elastic deformation region. A higher elastic modulus denotes that the film is stiffer. For the same thickness (0.6 mm), the 50% humidity sample contains approximately 3% water after equilibrium, and the 75% humidity sample contains approximately 9% water. According to previous studies, the more water contained in the films, the lower the elastic modulus of the films. As shown in Figures 6–9 and Tables 5–7, there is an obvious trend that the vacuum dry samples have the highest elastic modulus at 830 Mpa, and the 70% humidity samples have the lowest elastic modulus at 711 Mpa. Compared with vacuum-dried samples, the elastic modulus of 50% humidity samples decreases by 12.5%. However, the elastic modulus of 70% humidity samples only decreases by 2.1% compared with that of 50% humidity samples. The water content in the 70% humidity samples is 6% more than that of the 50% humidity samples. It is concluded that environmental humidity can largely influence the elastic modulus of the soaked Mori films, and the impact of the environmental humidity on the elastic modulus of soaked Mori films is not linearly correlated. To figure out the impact of humidity on the thermal properties of the samples, DSC was performed, as shown in Supplementary Figure S4. It can be concluded that humidity did not affect the thermal properties of the composites as they all show a degradation temperature of approximately 261°C. However, the higher the humidity, the more energy was required to degrade the sample.

**TABLE 5:** Mechanical properties of three Mori films after reaching the equilibrium in the vacuum chamber with 30 in Hg vacuum, including elastic modulus, ultimate strength, and maximum elongation at break.

Samples	Maximum elongation at break (%)	Ultimate strength (MPa)	Elastic modulus (MPa)
Vacuum dry_1	4.72	25.47	887
Vacuum dry_2	4.42	16.37	783
Vacuum dry_3	5.06	25.58	821
Average	4.73 ± 0.32	22.47 ± 5.29	830 ± 53

**TABLE 6:** Mechanical properties of three Mori films after reaching the equilibrium in the humidity chamber with 50% humidity, including elastic modulus, ultimate strength, and maximum elongation at break.

Samples	Maximum elongation at break (%)	Ultimate strength (MPa)	Elastic modulus (MPa)
50% Humidity_1	4.12	30.35	768
50% Humidity_2	7.37	35.89	679
50% Humidity_3	6.42	42.63	732
Average	5.97 ± 1.67	36.29 ± 6.14	726 ± 45

**TABLE 7:** Mechanical properties of three Mori films after reaching the equilibrium in the humidity chamber with 70% humidity, including elastic modulus, ultimate strength, and maximum elongation at break.

Samples	Maximum elongation at break (%)	Ultimate strength (MPa)	Elastic modulus (MPa)
70% Humidity_1	4.85	36.31	785
70% Humidity_2	5.43	33.36	699
70% Humidity_3	7.76	37.13	647
Average	6.01 ± 1.54	35.6 ± 1.98	711 ± 70

We conducted short-term mechanical properties of SF film samples with varying thicknesses and SF films that were compressed in the wet state. The samples were soaked, dried, and kept in the humidity chamber at room temperature (∼25°C) with a 50% humidity level before testing. We also performed the mechanical stress–strain tensile tests for SF films that have been hot-pressed, rolled, and fixed uniaxially in the x- or y-direction during drying to understand the film geometry, processing method, and environmental impacts on their mechanical properties. From the stress–strain curves, we calculated the mechanical parameters such as elastic modulus, strength of extension, and elongation ratio. Similar to wet pressing, hot pressing is a process by which the material is placed between two plate metals and compressed to form a flat mold that is in the form of a sheet but subjected to a temperature of 100°C for 15 min under a load of 100 KN. Hot pressing of thermoplastic polymers is a common process used to fabricate high precision, high-quality features at the micro/nanoscale. This method is applied in industrial production. Other modes of pressing can include roll-to-plate and roll-to-roll (R2R) and can be used to pattern the SF films, if necessary. All samples were dried, fixed in either the x- or y- (longitudinal and transverse) direction, and kept at room temperature (∼25°C) with a humidity level of 50% for 4 days before the mechanical tests. The samples were tested at least five times to obtain a statistical error bar.

### 3.6 Effects of different processing methods

**FIGURE 10:**
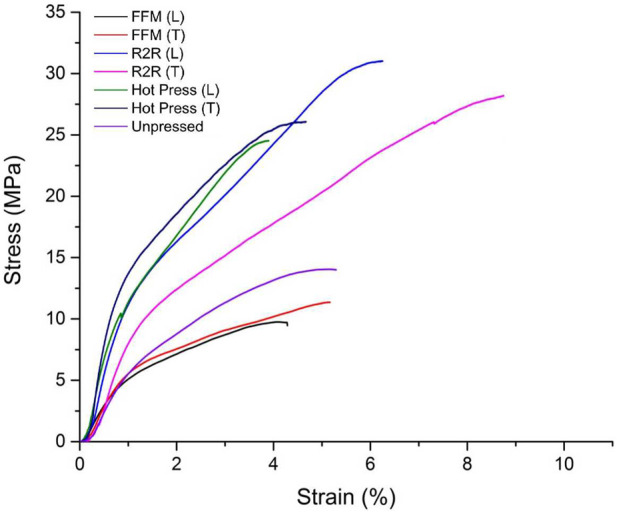
Stress–strain curves for parameters for FFM, R2R, hot-pressed, and uncompressed samples in the x- and y-directions after humidity conditioning.

In Figure 10, the tensile behavior (stress–strain curves) of FFM, rolled, and hot-pressed SF films in the x- and y-direction, moisturized at 50% relative humidity and 25°C, is shown. Supplementary Figure S5 shows the scheme of FFM, and Mori silk films dried by the FFM fixture are shown in Supplementary Figure S6. Changes in tensile strength, Young’s modulus, and maximum elongation are shown in Table 8. The current study indicates that the fixation of SF affects the tensile properties in the transverse direction, showing a slight increase in elastic modulus and elongation compared to films fixed in the longitudinal direction. The fixed transverse film also closely resembles the uncompressed SF samples.

**TABLE 8:** Mechanical parameters for FFM, R2R, hot-pressed, and uncompressed samples in the x- and y-directions, including elastic modulus, ultimate strength, and maximum elongation at break.

SF film	Maximum elongation at break (%)	Ultimate strength (MPa)	Elastic modulus (MPa)
Fixing process (transverse)	6.1 ± 1.4	12 ± 0.6	280 ± 110
Rolling process (longitudinal)	6.1 ± 0.7	30 ± 2.4	680 ± 190
Rolling process (transverse)	8.8 ± 1.2	30 ± 3.4	590 ± 130
Hot press (longitudinal)	4.1 ± 1.3	22.5 ± 4.3	950 ± 270
Hot press (transverse)	4.5 ± 0.9	30 ± 4.1	940 ± 250
Unpressed	6.6 ± 1.1	14 ± 1.1	320 ± 130

There are two types of compression processes, namely, cold temperature compression and hot compression. The hot press machine is shown in Supplementary Figure S7. Previously, in wet pressing, 60% wet sample films were pressed into sheets at room temperature. For hot compression molding, a hot press at a temperature of 100°C with a load of 100 KN was applied by pumping the pressure screw assembly lever to press the film. The sample was put on 5 × 5 mm upper and lower square platens and compressed for 15 min. The applied heat allows the 60% soaked film to dry and crystallize rapidly into a flat sheet. The flat film is then cut to a width of 10 mm and a gauge length of 40 mm, fixed in the x- or y-direction, and conditioned in a humidity chamber. In Figure 10, the tensile behavior (stress–strain curves) of hot-pressed SF films in the x- and y-direction is shown. Changes in tensile strength, Young’s modulus, and strain at yield versus moisture are shown in Table 8. The current study indicates clearly that the net impact of compression on the tensile properties of SF is increased. At 10 KN tons with a temperature of 100°C, changes in tensile behavior increase the ultimate tensile strength and enhance the elastic modulus after pressing, but the total elongation (or strain) at failure remained virtually unchanged. Fixing the SF films after pressing in the longitudinal and transverse directions did not enhance or affect the already hot-compressed samples.

For the roll processing, soaked SF films were rolled biaxially to 0.26 mm thickness reductions at 60°C. The rolling process is a continuous process that transfers a flexible substrate through two moving rolls of material. This manufacturing technique provides a process for efficiently producing a biaxially oriented SF film. This can also produce dense, thin films by layer by layer (LBL) lamination with high efficiency and yield under wet conditions. As previously described, the production process comprises a casting–drying step of dissolving SF onto the surface of a rectangular PE mold. After air drying, the SF material is soaked for 24 h and pat-dried with a towel. The unstretched sheet is then fed into the roll-to-roll press. The R2R press allows biaxial stretching to occur, extending the unstretched film sheet in longitudinal and transverse directions. The films were cut into appropriately sized rectangular and dog-bone specimens with a width of 12.5 mm and a gauge length of 40 mm before fixing the specimens in the x- or y-direction. After humidity conditioning, the tensile properties of the rolled and unrolled SF sheets were measured both in the longitudinal and transverse directions at room temperature. In Figure 5, the tensile behavior (stress–strain curves) of rolled SF films in the x- and y-directions is shown. Changes in tensile strength, Young’s modulus, and strain at yield versus moisture are shown in Table 4. It is found that the tensile strength of the material is enhanced with the rolling. Fixing the SF films after rolling in the longitudinal and transverse directions can slightly increase the percent elongation.

Additionally, the rolling process can be investigated using the soaked SF film as a crystalline polymer at various rolling temperatures, examining the rolling characteristics, cross-section morphologies, and tensile properties. FFM, R2R, and hot compression are important manufacturing processes that continuously produce thin films. Further studies on hot compression molding may lead to crystal setting matrices. With these thin-film processing techniques, protein-based thin films can be transferred from the laboratory to a more production-oriented environment. To figure out how the process method affects the secondary structures of silk fibroin, FT-IR analysis was performed, as shown in Supplementary Figure S8. It can be found that the peak in the amide I area of each sample shifted from 1,641 cm^−1^ for unpressed samples to 1,620 cm^−1^ for FFM samples, which suggests that the β-sheet content was increased after hot-pressed and fixed edge drying; thus, the mechanical properties were also modified using different methods.

The crystalline-to-amorphous phase ratios and β-sheet content critically govern the mechanical behavior of RSF films, as demonstrated by FT-IR analysis (Supplementary Figure S8) and tensile testing (Tables 4, 8; Figures 5, 10). For hot pressing (FFM), an increase in β-sheet content (amide I peak shift from 1,641 cm^−1^ to 1,620 cm^−1^) correlates with enhanced stiffness and strength but reduced ductility. This is because compression-induced alignment of fibroin chains promotes β-sheet crystallization, creating rigid networks that resist deformation. For plasticizer incorporation, the amorphous phase dominated the mechanical properties. Reduced β-sheet content increases free volume, enabling increased elongation at break but reduced modulus. Small-molecule plasticizers (e.g., glycerol) disrupt H-bonding, while polymeric plasticizers (e.g., PVA) form semi-IPNs, balancing flexibility and strength. As for humidity treatments, low humidity favors β-sheet formation, yielding high modulus but brittleness. High humidity induces amorphous hydration layers, increasing ductility at the expense of strength. As for roll-to-roll (R2R) processing, biaxial stretching increases crystalline orientation, enhancing anisotropic strength and modulus. These trends confirm that crystalline phases (β-sheets) drive stiffness and strength, while amorphous regions govern ductility. Processing parameters (temperature, pressure, and humidity) systematically tune this balance, enabling RSF films to mimic tissues ranging from flexible skin (low crystallinity) to stiff tendons (high crystallinity).

## 4 Conclusion

This research represents a comprehensive and systematic exploration into the modulation of the mechanical properties of RSF materials through the incorporation of plasticizers and the application of diverse treatment modalities. The addition of plasticizers has been demonstrated to exert a profound influence, significantly enhancing the elongation at break of RSF films while maintaining an elastic modulus commensurate with that of human tissues. This outcome is of particular significance as it endows the RSF materials with improved flexibility and deformability, thereby enhancing their potential for compatibility with biological systems. Conversely, the implementation of distinct processing methods, such as solvent treatments, environmental humidity drying variations, FFM, and roll-to-roll processing, yields RSF materials with elevated elastic moduli. However, this enhancement is accompanied by a notable reduction in elongation at break. It is hypothesized that this phenomenon can be attributed to the extensive removal of water molecules from the RSF materials during these processes, which leads to a more compact and rigid aggregation of silk fibroin fibers. This study demonstrates that the mechanical properties of silk fibroin can be modulated by various methods, which can be used in different biomedical engineering-related scenarios.

## Data Availability

The original contributions presented in the study are included in the article/Supplementary Material; further inquiries can be directed to the corresponding authors.
