# Supplementary material for: Engineering the mechanical characteristics of regenerated silk fibroin materials: the impact of chemical and physical modification strategies
Source: Front Chem. 2025 Jun 10;13:1606995. doi: 10.3389/fchem.2025.1606995 (PMC12185429; doi:10.3389/fchem.2025.1606995)
Supplement: Supplementary file 1 [file Supplementaryfile1.docx]

Supplementary Material


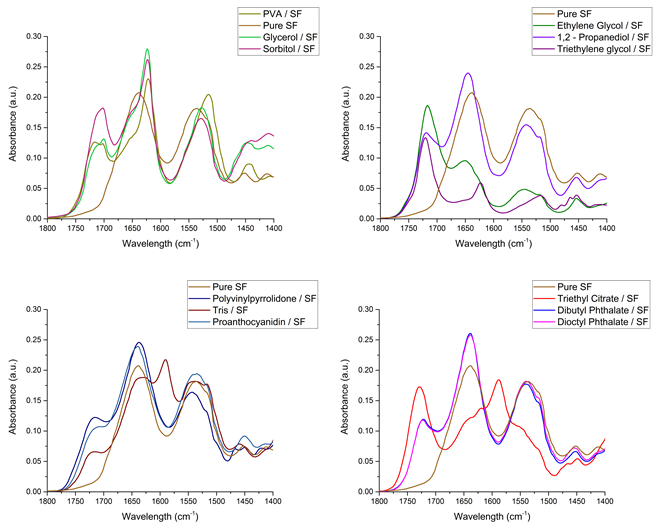


**Figure S1**. FTIR analysis of different SF plasticized films.

**Table S1**. Properties of silk films blended with various plasticizers.

| SF Films  Plastizers | Polarity | Phase separation | Wt. (g) after casting/drying | Wt. (g) after soaking/drying |
| --- | --- | --- | --- | --- |
| Oleic Acid | hydrophobic | Yes | 1.3363 | 1.1107 |
| Triethyl Citrate | hydrophobic | No | 1.5529 | 1.0598 |
| Dibutyl Phthalate | hydrophobic | Yes | 1.4348 | 1.1494 |
| Dioctyl Phthalate | hydrophobic | No | 1.407 | 1.1459 |
| Ethylene Glycol | hydrophilic | Yes | 1.4303 | 0.5198 |
| Polyvinylpyrrolidone | hydrophilic | No | 0.98884 | 0.6019 |
| 1,2 - Propanediol | hydrophilic | Yes | 1.6574 | 0.6209 |
| Triethylene glycol | hydrophilic | No | 1.9101 | 1.0131 |
| Tris | hydrophilic | No | 0.9433 | 0.4719 |
| PVA | hydrophilic | No | 0.2165 | 0.2161 |
| Proanthocyanidin | hydrophilic | No | 0.9244 | 0.6525 |
| Proanthocyanidin/ GA | hydrophilic | No | 0.8086 | 0.8074 |
| Glycerol | hydrophilic | No | 0.9035 | 0.7063 |
| Sorbitol | hydrophilic | No | 0.9658 | 0.8324 |


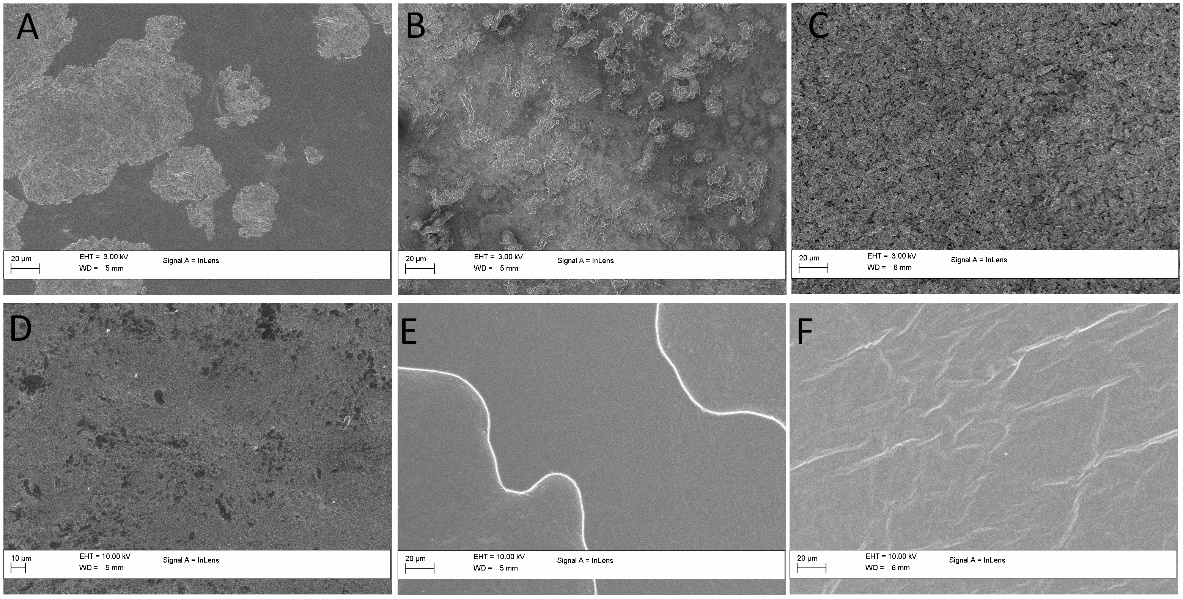


**Figure S2**. SEM top views of silk composite films: A. Silk-Tris (tris(hydroxymethyl)aminomethane); B. Silk-Triethyl Citrate; C. Silk-Triethylene glycol (TEG); D. Silk-Polyvinylpyrrolidone (PVP); E. Silk-Dioctyl Phthalate (DOP); F. Silk-Oleic Acid.


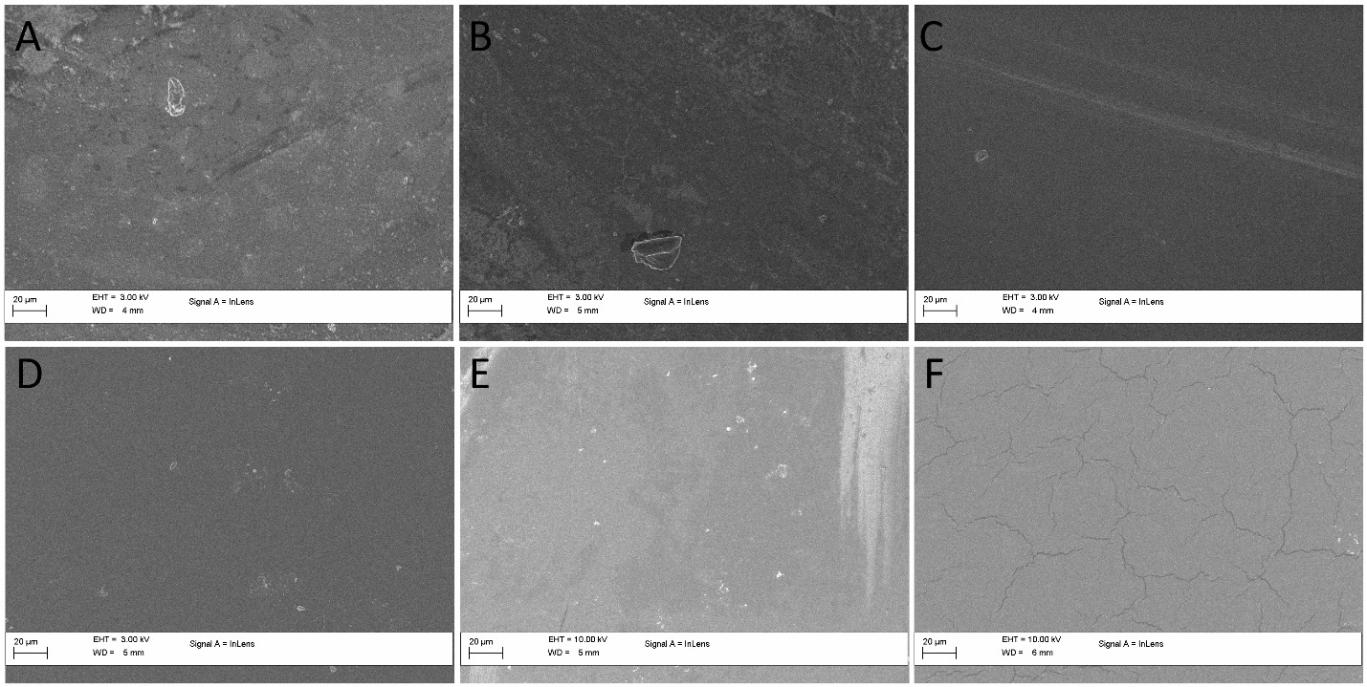


**Figure S3**. SEM top views of silk composite films: A. Silk-Glycerol; B. Silk-Sorbitol; C. Silk-Poly(vinyl alcohol) (PVA); D. Silk-Proanthocyanidin; E. Silk-Dibutyl Phthalate (DBP); F. Silk-Glutaraldehyde.

**Figure S4.** DSC curves of Vacuum dry sample and 50% Humidity sample.


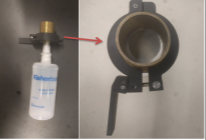

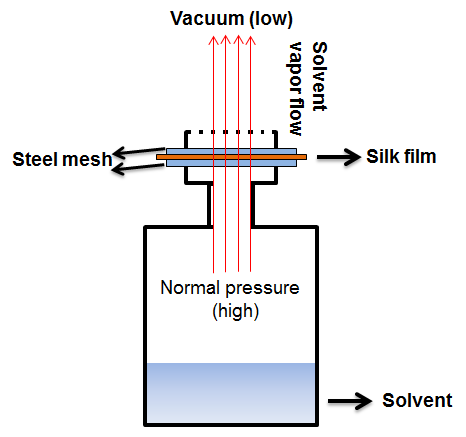


**Figure S5.** A scheme showing the FFM.


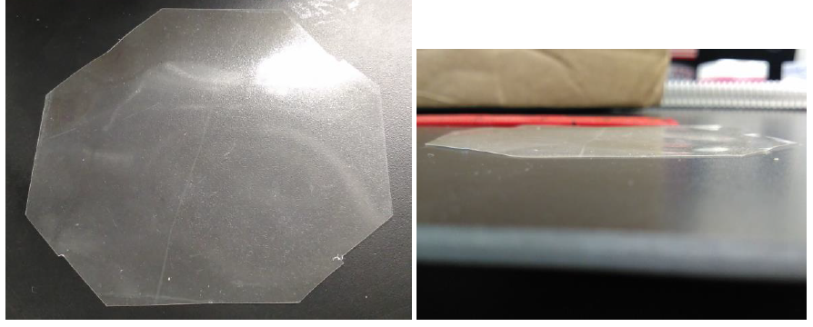


**Figure S6.** Mori silk film dried by the FFM fixture

**Figure S7.** Hot pressing machine (YLJHP88V, MTI Corporation)


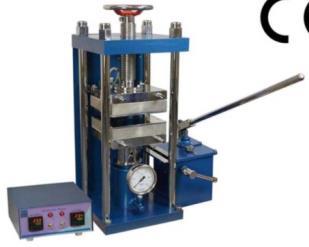

**Figure S8.** FTIR curves of Unpressed sample, Hot Press sample and FFM.
